# Cancer-Associated Fibroblasts Regulate Bladder Cancer Invasion and Metabolic Phenotypes through Autophagy

**DOI:** 10.1155/2021/6645220

**Published:** 2021-05-25

**Authors:** Dahai Dong, Yu Yao, Jinlei Song, Lijiang Sun, Guiming Zhang

**Affiliations:** Department of Urology, The Affiliated Hospital of Qingdao University, Qingdao, China

## Abstract

Recently, both cancer-associated fibroblasts (CAFs) and autophagy have been proven to play an important role in tumor development, including bladder cancer (BCa). However, the real mechanisms remain largely unclear. Here, we reconstruct a mimic tumor microenvironment to explore the interaction between CAFs and the BCa cell line T24 using a coculture system. Autophagy in CAFs was induced or inhibited by rapamycin or siRNA, respectively. After coculture with CAFs, T24 cell proliferation, invasion, and aerobic glycolysis were tested *in vitro*. Rapamycin induced and siAtg5 inhibited autophagy in CAFs. Enhanced autophagy in CAFs promoted cell proliferation and invasion in T24 cells in vitro, while there was no significant difference between the autophagy-inhibited group and the controls. Lactate concentration was elevated in both rapamycin-treated and siAtg5-treated groups compared with the control group. In addition, the expression levels of MCT1, MCT4, HK2, SLC2A1, and MMP-9 were all increased in T24 cells in the autophagy-enhanced group. Our results indicated that CAFs could regulate BCa invasion and metabolic phenotypes through autophagy, providing us with new alternative treatments for BCa in the future.

## 1. Introduction

Bladder cancer (BCa) is the sixth most common malignancy in males and the ninth when both genders are included worldwide, with an age-standardized incidence rate of 9.0 for males and 2.2 for females (per 100,000 persons/year) [1]. It causes an enormous economic burden, impairs patients' life quality, and threatens victims' survival when it invades the muscle. Even in those with a non-muscle-invasive disease, the BCa patients suffer from a high recurrence rate after surgical removal [2]. Both patient genetic background and environmental factors contribute to bladder carcinogenesis. Indeed, tobacco smoking and occupational exposure to chemicals are best-known environmental risk factors [3]. Besides, recent studies have reported that metabolic syndrome, herbs containing aristolochic acids, and pioglitazone treatment were associated with an increased risk of BCa [4–6]. However, despite advances in etiology seeking and therapeutic strategy, the real disease mechanisms and feasible prevention measures, as well as the long-term oncological outcomes, still remain limited.

A tumor is a complex structure comprising multiple cell types. Cancer cells are surrounded by stromal cells, such as endothelial cells, fibroblasts, and immune cells, which create and maintain the tumor microenvironment. Through complicated cross-talk and feedback, cancer cells interact with these stromal cells, which are capable of promoting or reversing the natural development of cancer. In recent years, the tumor microenvironment has received renewed attention as a critical determinant of cancer pathogenesis, likewise as a strong predictor of clinical outcome. For example, fibroblasts can be activated by cancer cells and transformed into cancer-associated fibroblasts (CAFs) [7], and paracrine communication between CAFs and cancer cells has been proven to be crucial for the continued growth of cancer mass [8]. Previous studies have found that CAFs accelerate tumor growth and metastasis via oxidative stress and aerobic glycolysis [9]. Oxidative stress in CAFs can drive tumor-stroma coevolution, and metabolites resulting from aerobic glycolysis of CAFs can be used by cancer cells to promote energy production [10, 11]. Our previous study also observed that overexpression of monocarboxylate anion transporters (MCT) 1 and 4, two key biosynthetic transporters involved in lactate transportation, in CAFs could regulate the progression of BCa cells [12]. Therefore, deeply understanding the ways by which cancer cells interplay with CAFs will provide a new insight into tumor initiation and development.

Autophagy is a major intracellular degradation system that aids with the turnover of long-lived proteins and damaged organelles [13]. During autophagy, proteins and/or organelles are encased by double-membrane vesicles termed autophagosomes, which ultimately fuse with the lysosomal compartments for protein degradation to take place. Since it links transformed and nontransformed components of the tumor microenvironment, autophagy is considered to be important for cancer initiation, progression, and response to therapy [14]. Autophagy can be either tumor suppressive or tumor promoting. Ojha et al. observed much more autophagic vesicles in BCa tissues than in adjacent benign bladder tissues, and higher levels of Beclin-1 and Atg7 in tumor specimens [15]. Mani et al. demonstrate that the chemotherapy-resistant BCa cell lines utilized autophagy to escape apoptotic cell death [16]. In recent years, several research groups reported that autophagy in CAFs might be involved in the development of breast cancer. They found that autophagic CAFs could play a cancer-supportive role by preventing the death of adjacent cancer cells [10, 17]. However, little information is known about the role of autophagy in fibroblast-BCa cell interaction. Here, using a coculture system, we show that fibroblasts could regulate BCa invasion and metabolic phenotypes through autophagy, and the results may offer new alternative treatments for BCa in the future.

## 2. Materials and Methods

### 2.1. Reagents

The primary and secondary antibodies were as follows: AKT and phosphorylated AKT (p-AKT) (Ser473) (Cell Signaling Technology, Boston, MA); Beclin-1, MCT1, MCT4, HK2, and SLC2A1 (Abcam, Cambridge, MA); LC3-I/II and Atg5 (Sigma-Aldrich, St. Louis, MO); CD34 (Boster, Wuhan, China); and mTOR, phosphorylated mTOR (p-mTOR), MMP-9, GAPDH, and horseradish peroxidase- (HRP-) conjugated secondary antibodies (goat anti-mouse IgG HRP and goat anti-rabbit IgG HRP) (Santa Cruz Biotechnology, Dallas, TX).

### 2.2. Cell Culture

The human BCa cell line T24 and human foreskin fibroblast (hFF) cells were obtained from the Institute of Cell Research of the Chinese Academy of Sciences (Shanghai, China). All cells were maintained in RPMI1640 medium containing 10% fetal bovine serum (FBS) (Gibco, Grand Island, NY) and 1% antibiotics (HyClone, South Logan, UT) and cultured at 37°C at 5% CO_2_.

**Figure 1:**
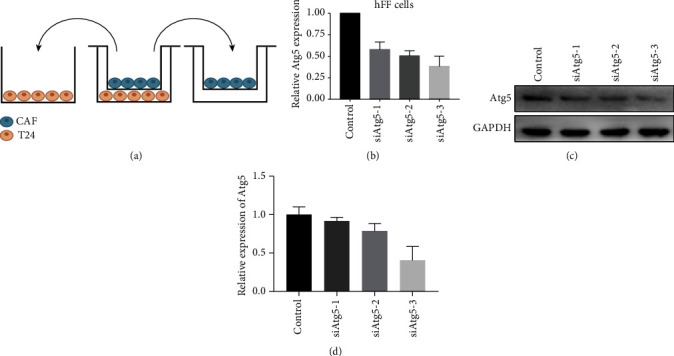
Downregulation of Atg5 in hFF cells. (a) Schematic representation of a Transwell coculture system: hFF cells were seeded in the upper chamber of a 6-well Transwell and T24 cells were in the bottom chamber (pore size: 0.4 *μ*m). After 48 hrs, T24 cells were trypsinized acquired for further experiments; the effects of Atg5 siRNA on hFF cells were detected by RT-PCR (b) and Western blot (c, d). Because of the high efficiency in decreasing Atg5 expression, siAtg5-3 was chosen for further experiments.

To mimic the BCa epithelial-stromal interaction in the tumor microenvironment, T24 cells and hFF cells were cocultured in a Transwell cell culture system (Figure 1(a)). In brief, hFF cells were seeded into the upper chamber (pore size 0.4 *μ*m) and cocultured with T24 cells in the lower chamber in 6-well Transwell plates. After 48 hrs, T24 cells were trypsinized acquired for further experiments. Through this process, we have previously demonstrated that hFF cells could be induced to be CAFs [12].

### 2.3. Cell Proliferation and Invasion Assays

T24 cell proliferation was determined using the CCK-8 (Cell Counting Kit-8) assays. Briefly, cells were seeded in 96-well plates. After culture overnight, the medium in each plate was replaced by fresh medium containing 10% CCK-8 reagent (Dojindo Molecular Technologies, Shanghai, China) according to the manufacturer's protocols. Then, cells were incubated for 2 hrs and evaluated with a microplate reader at 450 nm.

T24 cell invasion was assessed using the Transwell assays. Briefly, cells were seeded in the upper chamber coated with 60 *μ*l Matrigel (BD Biosciences, Franklin Lakes, NJ) diluted with serum-free medium (1 : 50). The lower chamber was filled with medium containing 10% FBS. Following incubation for 48 hrs, cells were fixed using 75% ethanol and stained using 0.5% crystal violet. Invaded cells were observed using an inverted microscope, and numbers were counted in three randomly selected microscopic fields.

### 2.4. siRNA Transfection, RNA Extraction, and Quantitative Real-Time Polymerase Chain Reaction (qRT-PCR)

The sequence of Atg5-targeted siRNA that was chosen out of three pairs of siRNAs (GenePharma, Shanghai, China) because of its high efficiency in decreasing Atg5 expression was 3′-CGACGACGTTCGGTTCCGCCGTC-5′. siRNA-mediated Atg5 knockdown was performed using Lipofectamine 2000 (Invitrogen, Carlsbad, USA) according to the manufacturer's instruction. Briefly, hFF cells were seeded in 6-well plates and cultured overnight. The diluted Lipofectamine 2000 and siRNA were mixed at room temperature for 20 min and then were added into the plate. After incubation at 37°C for 6 hrs, the medium was refreshed and followed by continuous culture for 48 or 72 hrs. Then, the transfected cells were harvested for further experiments.

Total cell RNA was isolated using TRIzol reagent (Invitrogen, Carlsbad, CA) and then converted into cDNA using the RevertAid First Strand cDNA Synthesis Kit (Life Technologies, Carlsbad, CA) according to the manufacturer's protocol. qRT-PCR was carried out by the Power SYBR Green PCR Master Mix (Invitrogen, Carlsbad, CA) in an ABI 7900HT Real-Time PCR System. All tests were performed in triplicate with two sets of independent experiments, and *β*-actin was used as an internal control. The primer sequences were listed as follows: Atg5—forward: 5′-TTCTCAAAATATACTGTTTC-3′, reverse: 5′-TATTATGTATCACAAATGG-3′; *β*-actin—forward: 5′-ACCGAGCGCGGCTACAG-3′, reverse: 5′-CTTAATGTCACGCACGATTTCC-3′.

### 2.5. Lactate Measurement

According to the manufacturer's protocol, EnzyChrom™ Lactate Assay kit (ECLC-100) (BioAssay Systems, Hayward, CA) was used to determine lactate concentration in cell culture medium. Briefly, the standard curve was drawn based on a series of lactate solutions with known concentrations. Cells were cultured for 48 hrs, and 20 *μ*l of media was collected and measured by spectrophotometry at 565 nm. Then, results were normalized for total cell number.

### 2.6. Western Blot

The procedure was described previously [18]. In brief, after the transfection for 72 hrs, T24 cell and hFF cell protein was extracted using the CelLytic extraction kit supplemented with protease inhibitors (Roche, Basel, Switzerland). The total protein concentration was measured using the BCA Protein Assay reagent kit (Thermo Fisher Scientific, Waltham, MA) according to the manufacturer's instruction. Equal amounts of cell lysate were electrophoresed on a 12% polyacrylamide gel, and the protein was transferred to an Immobilon-P PVDF membrane. Then, nonspecific binding was blocked at room temperature with 5% defatted milk. The membrane was then incubated with primary antibodies at 4°C overnight, followed by incubation at room temperature with a second antibody for 1 hr. Signals were detected by the ECL Plus Western Blotting System (Thermo Fisher Scientific, Waltham, MA).

### 2.7. Immunofluorescence

hFF cells were seeded on microscopic slides overnight and fixed with 2.5% glutaraldehyde for 20 min. After incubating in 1% BSA and 0.25% Triton X-100 in PBS, cells were incubated with CD34 antibody at 4°C overnight, followed by incubation with FITC green-conjugated secondary antibody (Boster, Wuhan, China) for 1 h at room temperature. Nuclei detection was performed using DAPI costaining. Fluorescence images were acquired with a laser confocal microscope.

### 2.8. Statistical Analysis

Data are expressed as mean ± standard deviation (SD). Difference between groups was compared with Student's *t*-test, and significance was established at *P* < 0.05. All statistical analyses were carried out using STATA 12.0.

## 3. Results

### 3.1. Autophagy in hFF Cells Is Induced or Inhibited by Rapamycin or siAtg5, Respectively

We downregulated the expression of Atg5 in hFF cells by siRNA. As shown in Figure 1, both mRNA (Figure 1(b)) and protein levels (Figures 1(c) and 1(d)) of Atg5 were obviously decreased using siAtg5-3; thus, we chose it for further experiments.

**Figure 2:**
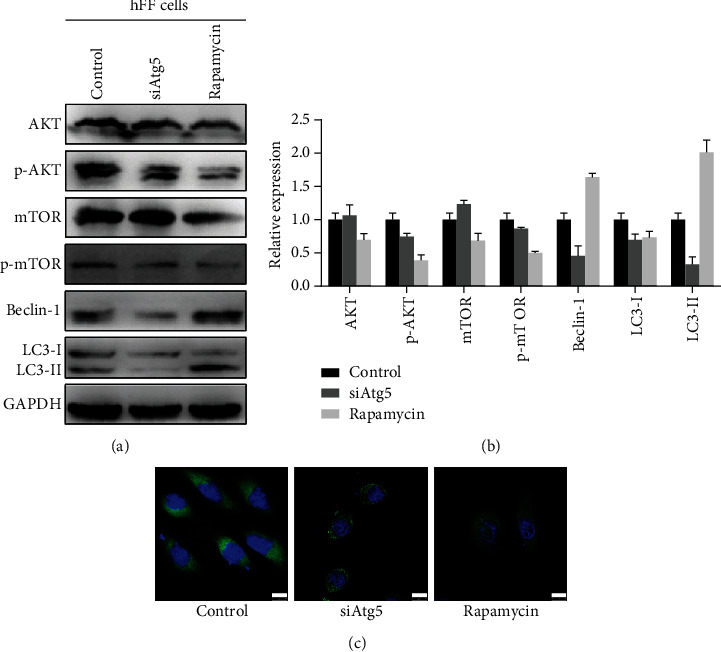
(a, b) Autophagy in hFF cells was induced or inhibited by rapamycin or siAtg5, respectively, and detected by Western blot: Beclin-1 and LC3-II/I levels were both increased after the induction by rapamycin, while decreased after the inhibition by siAtg5. Additionally, rapamycin administration inhibited the expression of AKT/p-AKT/mTOR/p-mTOR, while siAtg5 treatment took little effect on the expression of AKT/mTOR and their phosphorylation levels; (c) immunofluorescence imaging indicated that CD34 expression (green) in hFFs was downregulated in rapamycin- or siAth5-treated groups compared with the control group; DAPI was used to visualize nuclei (blue).

We used rapamycin to induce and siAtg5 to inhibit autophagy in hFF cells. As shown in Figures 2(a) and 2(b), Beclin-1 and LC3-II/I levels were both increased after the induction by rapamycin, while decreased after the inhibition by siAtg5. In addition, the AKT/mTOR signaling pathway serves an essential role in the regulation of autophagy; hence, we detected the expression of AKT, mTOR, and their phosphorylation levels in hFF cells. Rapamycin, an inhibitor of mTOR, inhibited the expression of AKT/p-AKT/mTOR/p-mTOR, while siAtg5 treatment did not take obvious effect on the expression of AKT/mTOR and their phosphorylation levels.

### 3.2. Autophagy in CAFs Affects T24 Cell Growth and Invasion In Vitro

**Figure 3:**
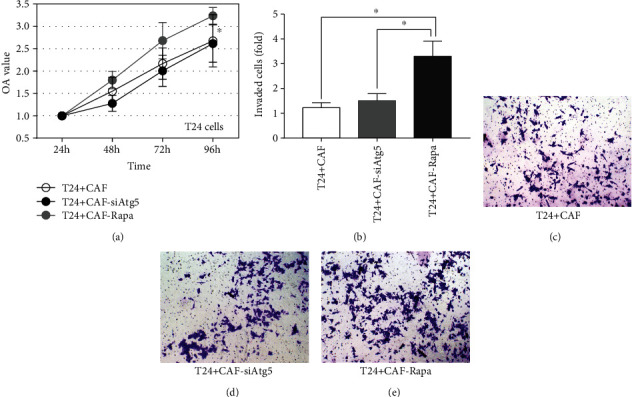
Autophagy in CAFs affects T24 cell growth, invasion, and aerobic glycolysis *in vitro*: after coculture with CAFs (autophagy was induced or inhibited), T24 cell proliferation was tested using the CCK-8 assays (a) and cell invasion was tested using the Transwell assays (b–e) (^∗^*P* < 0.05).

**Figure 4:**
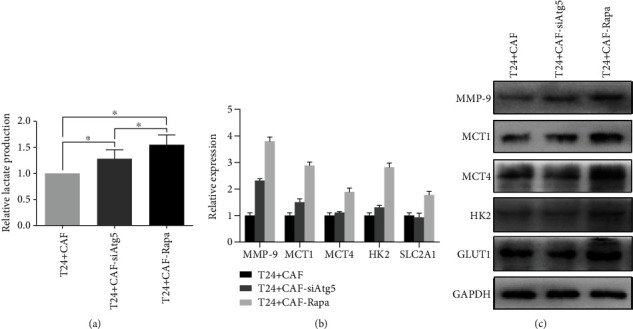
Autophagy in CAFs affects aerobic glycolysis and MMP-9 expression in T24 cells in vitro: (a) compared with the control group, the lactate levels in the cell medium increased in both siAtg5-treated and rapamycin-treated groups (^∗^*P* < 0.05); (b, c) Western blot analyses of MMP-9 and several key glycolytic genes, including HK2, SLC2A1, MCT1, and MCT4 in T24 cells: the expression levels of these genes increased obviously in the autophagy-enhanced group.

We have previously demonstrated that coculture with T24 cells could activate hFF cells into CAFs (12). In this study, we also observed that T24 cells can also activate rapamycin- or siAth5-treated hFFs into CAFs. In addition, CD34, which is a common marker for diverse progenitors, was downregulated in the experimental groups compared with the control group (Figure 2(c)). Then, we further investigated whether autophagy in CAFs affected T24 cell growth and invasion in the tumor microenvironment. As indicated in Figure 3(a), rapamycin-treated CAFs promoted T24 cell viability compared with the controls (*P* < 0.05), while inhibiting autophagy in CAFs did not influence T24 cell proliferation. Likewise, autophagy in CAFs enhanced the ability of invasion in T24 cells in vitro (*P* < 0.05), while there was no significant difference between the siAtg5-treated group and the controls (Figures 3(b)–3(e)). In BCa, MMP-9 has a positive correlation with tumor progression and invasion [19]. Thus, we detected the expression levels of MMP-9 and observed increased protein expression in the autophagy-induced group (Figures 4(b) and 4(c)).

### 3.3. Autophagy in CAFs Affects Aerobic Glycolysis in T24 Cells In Vitro

To better understand whether autophagy in CAFs can regulate BCa aerobic glycolysis, we detected lactate levels because lactate is the end-product of glycolysis in the hypoxic tumor cell compartment. As shown in Figure 4(a), lactate concentration was elevated in both rapamycin-treated and siAtg5-treated groups compared with the control group (*P* < 0.05). Enhanced autophagy in CAFs resulted in a much higher level of glucose usage of T24 cells. Furthermore, we detected several key glycolytic genes that participate in glucose metabolism, including MCT1, MCT4, HK2, and SLC2A1. As expected, the expression levels of these four genes increased in the autophagy-enhanced group (Figures 4(b) and 4(c)).

## 4. Discussion

Tumors are complex organ facsimiles that comprise not only neoplastic cells but also stromal cells and matrix components [20]. To maintain the homeostasis of the tumor microenvironment, different cell types interplay with each other and with the extracellular matrix components that provide the structure of tumor architecture. CAFs are important components in this tumor microenvironment, playing a central role in the modulation of cancer cell growth. They can secret some important soluble factors, such as transforming growth factor beta 1 (TGF-*β*1), and create a favorable milieu for cancer cells to grow and invade [21]. Yeh et al. reported that a higher expression of estrogen receptor-*α* in fibroblasts may promote BCa invasion through enhancing the CCL1 and IL-6 signals in the tumor microenvironment [22]. In addition, recent studies have found that CAFs can create a nutrient-rich microenvironment via the local stromal generation of mitochondrial fuels, such as lactate, glutamine, and fatty acids, to metabolically support cancer development, and HIF1-*α* serves as a tumor promoter in CAFs in this metabolic coupling between CAFs and cancer cells [23, 24]. In the present study, we observed that augmented autophagy in CAFs promoted T24 cell proliferation and invasion in vitro and affected cell aerobic glycolysis. Our previous study also observed downregulation of MCT1 and MCT4 (two key genes involved in the lactate transportation) in CAF-suppressed T24 cell proliferation and decreased the concentration of lactate in the culture medium [12]. Lactate is the keystone of an exquisite symbiosis in which glycolytic and oxidative tumor cells mutually regulate their access to energy metabolites [25]. Therefore, these observations further reveal the mechanisms by which CAFs function as a crucial regulator in the tumor environment, facilitating a deeper understanding of the metabolic ecology of cancer.

Cancer cells induce the “Warburg effect” in neighboring stromal fibroblasts, and these fibroblasts secret lactate and pyruvate which are taken up by cancer cells for energy utilization [10]. This energy cross-talk boosts tumor growth and invasion. In addition, cell survival also depends on autophagy. Besides being responsible for the organelle turnover, autophagy also modulates cell metabolism and is considered as a tumor suppressor, particularly at the early stages of tumor initiation [26]. Indeed, a series of genes whose products are involved in autophagy are called autophagy-related genes (Atgs). Dependent on the complex formed by Beclin-1 and PI3Ks, which is localized in the trans-Golgi network, the isolated membranes of autophagosomes complete the process of nucleation and assembly. In addition, the expansion and closure of the autophagosome need two ubiquitin-like conjugation systems: LC3 and the Atg5-Atg12 complex. In an Atg4-dependent process, a C-terminal glycine residue is exposed in LC3, which is employed by Atg7 and Atg3 to conjugate phosphatidylethanolamine to LC3-I to form LC3-II [27]. Beclin-1, Atg4, Atg5, and Atg7 have indicated a tumor-suppressive function. mTOR is also a master regulator of cellular metabolism. It regulates cell growth and proliferation in response to a wide range of cues and also plays a crucial role in regulating autophagy. It has been proven that mTOR may inhibit autophagy. [28] Recently, autophagy has drawn much attention: not only autophagic cancer cells but also autophagic stromal cells have been proven to play an important role in cancer research. Capparelli et al. reported that autophagy in CAFs supported breast cancer growth and metastasis via glycolysis and ketone production [29]. Hypoxia, oxidative stress, HIF1 induction, and NF*κ*B activation in the tumor stromal microenvironment were found crucial in the process where autophagic CAFs promoted tumor cell survival [17]. Through rapamycin administration, we enhanced autophagy in CAFs as indicated by increased levels of Beclin-1 and LC3. In addition, as an inhibitor of mTOR, we observed that rapamycin inhibited the expression of mTOR/p-mTOR as expected, as well as the Akt/p-Akt expression. The effect of rapamycin on AKT/p-AKT varies in different cells. For example, 24 hours of exposure in rapamycin could inhibit the phosphorylation of AKT in PC-3 cells, but not in HeLa cells [30]. We speculate cell heterogeneity might lead to the inconsistency. After coculture of autophagic CAFs with T24 cells, cell proliferation and invasion were both enhanced, along with increased expression of MCT1, MCT4, HK2, and SLC2A1, as well as elevated levels of lactate in the tumor microenvironment. Inhibited autophagy in CAFs did not lead to the expected result: lactate concentration in the media was also elevated in the siAtg5-treated group, although lower than the autophagy-induced group, and expression of glycolysis-related genes did not increase significantly. We speculated that other factors might be involved in the autophagy-related cross-talk between tumor cells and stoma cells. However, all these results still indicated a promoting role of autophagic CAFs in BCa progression. This metabolically coupled relationship between cancer cells and stromal fibroblasts may provide us a novel but challenging therapeutic option in the future: it is possible that through targeting components in tumor microenvironment, we may explore a new approach against tumor development.

We also observed increased expression of MMP-9 in autophagy-enhanced CAF-induced T24 cells. MMP-9 belongs to the matrix metalloproteinase family which is a zinc-dependent endopeptidase with proteolytic activity against extracellular matrix components [31]. High expression of MMP-9 has been found in many kinds of cancers [19, 31]. It has been reported that MMP-9 can regulate tumor growth and promote malignant cell invasion and is associated with a poor prognosis [32]. Malignant cells overproduce MMP-9 which can destroy the basement membrane and type IV collagen, resulting in the disruption of tissue architecture and function [33]. Therefore, high expression of MMP-9 often means an enhanced ability of tumor invasion. Through the Transwell assays, we found that autophagy in CAFs promoted T24 cell invasion *in vitro*. We speculate that increased MMP-9 expression might partly contribute to these observations. However, cancer invasion and metastasis is a complicated process in which multiple biological changes and factors are involved. In addition, we only used T24 cells in the present study. Clearly, further studies are needed in more cell lines to clarify the deeper mechanisms by which CAFs regulate BCa invasion and metastasis.

## 5. Conclusions

In summary, our study observed that CAFs affected T24 cell growth, invasion, and metabolic phenotype through autophagy. The results of this study indicated an important role of autophagy of CAFs in regulating BCa biological behavior, providing a new insight into the complicated interaction between cancer cells and stromal cells in the tumor microenvironment. Alteration of autophagy in CAFs might be a new attractive metabolic target for tumor treatment.

## Data Availability

The data used to support the findings of this study are included within the article.
